# Nemo-Like Kinase in Development and Diseases: Insights from Mouse Studies

**DOI:** 10.3390/ijms21239203

**Published:** 2020-12-02

**Authors:** Renée Daams, Ramin Massoumi

**Affiliations:** Department of Laboratory Medicine, Translational Cancer Research, Faculty of Medicine, Lund University, 22381 Lund, Sweden; Renee.Daams@med.lu.se

**Keywords:** Nemo-like kinase (NLK), animal models, mice, development, immune system neuronal disorders

## Abstract

The Wnt signalling pathway is a central communication cascade between cells to orchestrate polarity and fate during development and adult tissue homeostasis in various organisms. This pathway can be regulated by different signalling molecules in several steps. One of the coordinators in this pathway is Nemo-like kinase (NLK), which is an atypical proline-directed serine/threonine mitogen-activated protein (MAP) kinase. Very recently, NLK was established as an essential regulator in different cellular processes and abnormal NLK expression was highlighted to affect the development and progression of various diseases. In this review, we focused on the recent discoveries by using *NLK*-deficient mice, which show a phenotype in the development and function of organs such as the lung, heart and skeleton. Furthermore, NLK could conduct the function and differentiation of cells from the immune system, in addition to regulating neurodegenerative diseases, such as Huntington’s disease and spinocerebellar ataxias. Overall, generations of *NLK*-deficient mice have taught us valuable lessons about the role of this kinase in certain diseases and development.

## 1. Introduction

### 1.1. Wnt Signalling Pathway

The Wnt proteins consist of conserved, secreted glycoproteins harbouring essential roles in the mechanisms directing cell proliferation, cell polarity and cell fate determination during embryonic development, as well as adult tissue homeostasis in various organisms [1]. Originally, the Drosophila Wnt protein Wingless (Wg) was identified in a mutagenesis screen for visual phenotypes in which mutations in Wg prevent the formation of the wing [2,3] while, in mice after cloning, int-1 as a proto-oncogene was found to be responsible to virally induce mammary tumours [4,5]. Since the protein Wg was shown to be homologous to the mouse Wnt1 proto-oncogene, the name Wnt was derived from combining Wingless and int-1 [6,7].

Extensive research has shown that Wnt signalling is a conserved pathway in metazoan animals. Wnt ligands stimulate multiple intracellular signalling cascades, such as the canonical Wnt/β-catenin and the non-canonical β-catenin independent pathways, which can be subdivided into the Wnt/Ca^2+^ and planar cell polarity pathways [8,9]. In canonical Wnt signalling, the absence of Wnt ligands result in phosphorylation of β-catenin bound by a destruction complex consisting of Axin, glycogen synthase kinase-3β (GSK-3β), casein kinase 1 (CK1) and adenomatous polyposis coli (APC). The phosphorylation of β-catenin facilitates its subsequent ubiquitination by β-TrCP [10] targeting β-catenin for proteasomal degradation. Upon binding of Wnt to its receptors Frizzled and LRP5/6 and phosphorylation of LRP5/6 by CK1 and GSK3β, the Dishevelled (Dvl) protein is recruited to this complex, which further inactivates the destruction complex and promotes accumulation of β-catenin in the cytoplasm. From the cytoplasm, β-catenin translocates to the nucleus where it forms an active complex with T-cell factor (TCF) and lymphoid enhancer factor (LEF) proteins and initiates target gene transcription [11,12].

### 1.2. Nemo-Like Kinase

Nemo-like kinase (NLK) is an evolutionary, conserved, atypical proline-directed serine/threonine mitogen-activated protein (MAP) kinase [13] and was originally identified in 1994 as the Drosophila gene nemo required for proper rotation of photoreceptor cells in the eye during ommatidia morphogenesis [14]. NLK is the murine homologue of Drosophila nemo that was first cloned in 1998 and was shown to bear sequence identity to human Cdc2 and mouse Erk-2. NLK also has an extended amino-terminal domain consisting of histidine, proline, alanine and glutamine [15]. In vertebrates, NLK is considered to be a negative regulator of the Wnt signalling pathway. Previous studies showed that NLK can regulate the transcriptional activity of the β-catenin-LEF/TCF complex by directly phosphorylating TCF4. This phosphorylation prevents DNA binding of the β-catenin-TCF4 complex and limits β-catenin-mediated target gene transcription through TCF/LEF [16,17]. Another mechanism of antagonising the Wnt/β-catenin signalling pathway by NLK is through activation of the transforming growth factor beta-activated kinase 1 (TAK1)-mediated non-canonical Wnt pathway and TAK1-binding protein 2 (TAB2). It was shown that TAB2 can directly interact with NLK as a scaffold protein, thereby enabling the interaction between NLK and TAK1 [18]. It has also been reported that NLK-associated RING finger protein (NARF), which is an E3 ubiquitin ligase, can form complexes with NLK. Activation of NLK caused phosphorylation of TCF/LEF and ubiquitination-mediated degradation of this complex by NARF [19]. NLK is known as a negative regulator of Wnt signalling in most studies; however, there are few studies in which NLK has been shown to positively promote the Wnt signalling pathway [20]. In zebrafish, NLK signalling contributed to midbrain tectum development by promoting neural progenitor cell proliferation [21]. Furthermore, it was shown that activation of NLK-induced phosphorylation of LEF1 and dissociation from histone deacetylase 1 (HDAC1) facilitated LEF1-mediated target gene transcription [21].

Besides being a regulator of Wnt signalling, NLK has been shown to regulate several other cell signalling pathways. Direct binding of NLK to STAT3 resulted in phosphorylation of STAT3 at Ser727, which requires the activity of the TAK1-NLK cascade [22,23]. Recently, it was found that NLK interacts with 14-3-3ζ (also known as YWHAZ) to repress the expression of E-cadherin by preventing dimerization of 14-3-3ζ and inhibiting the migration of non-small cell lung cancer cells [24]. NLK has the ability to inhibit NFκB signalling via IKK-complex and inhibition of p65 translocation [25]. Notch-dependent transcriptional activity requires the formation of a ternary complex consisting of Mastermind, Centromere-Binding Protein-1 (CBF-1), suppressor of hairless (Su(h)), DNA-binding protein LAG-1 (Lin-12 and Glp-1), and Notch Intracellular Domain (NotchICD). NLK is a negative regulator of Notch-dependent transcriptional activation by phosphorylating NotchICD and thereby decreasing the formation of this ternary complex [26].

Over the years, it has been discovered that NLK plays an important role in different cellular processes, such as cell migration, proliferation, apoptosis, and invasion, by affecting various downstream molecules. In addition, dysregulation in NLK expression can affect the development and progression of various diseases, including cancer, where NLK has been found to act as a tumour suppressor or oncogene depending on the tumour type [27]. For example, in colorectal cancer, NLK inhibits cell growth and promotes cell apoptosis via a p53-independent signalling pathway [28]. In breast cancer, NLK was found to be mainly localised in the nuclei of breast cancer cells compared to the cytosolic localisation in non-cancerous breast epithelial cells. The interaction between heat shock protein 27 (HSP27) and NLK mediated the nuclear localisation and protected the cancer cells from apoptosis [29]. Recently, it was shown that NLK expression in melanoma correlates with vascular endothelial growth factor receptor 2 (VEGFR2)-related microvessel formation and melanoma metastasis. NLK was found to be expressed in biopsied tissues of melanoma patients, but was expressed significantly lower in metastatic melanoma, implying that NLK may serve as a new prognostic marker [30]. In human laryngeal carcinoma, tissue down-regulation of NLK inhibited tumour cell proliferation and invasion, suggesting that NLK might be used as a potential molecular target for development and diagnosis of laryngeal carcinoma [31].

In this review, we have focused on the recent discovery in the field of NLK signalling by using mice as a model system.

## 2. NLK and Development

NLK was initially shown to be involved in various developmental processes, such as the promotion of mesoderm specification in sea urchins [32], mesoderm patterning, patterning of midbrain and forebrain in zebrafish [33], as well as oocyte maturation in Xenopus Laevis [34] and in mouse testes [35]. To explore the function of NLK in vivo, we generated an *NLK*-deficient mouse model by inserting a LacZ reporter gene upstream of exon 2 of the *NLK* gene, causing a frameshift mutation in the *NLK* gene resulting in the deletion of NLK in the entire mouse. These *NLK*-deficient animals showed a severe phenotype that died within 12–36 h after birth after becoming cyanotic before birth due to compromised lung development. The lungs of these mice showed smaller and compressed alveoli in addition to hyperthickening of the lung vasculature. This was due to the deletion of NLK that could not phosphorylated LEF1, which led to elevated levels of VEGF expression that caused aberrant proliferation of pulmonary epithelial and endothelial cells [36]. Furthermore, using mouse embryonic fibroblast (MEF) cells isolated from *NLK*-deficient animals, we could show an increase in cell proliferation and shortened cell cycle compared to wild-type cells. It was shown that activation of NLK can directly phosphorylate HDAC1, which in turn binds to LEF1 followed by repressed transcriptional activation of Wnt-target genes, including those supporting cell proliferation [37]. An inducible transgenic mouse with cardiac-specific NLK expression that was tetracycline-responsive caused significant damage in left ventricular chamber dilation and cardiac fractional shortening. These mice developed baseline cardiomyopathy and, with pressure overload, were more susceptible to heart failure. Furthermore, the cardiac tissue-specific transgenic knock-out model of *NLK* was protected from pathology associated with pressure overload and infarction injury [38]. The impact of NLK expression on skeletal development was studied in various transgenic mouse models. In the first model, conditional deletion of *NLK* in the limb buds of mice did not show any skeletal phenotype. In the second, conditional inactivation of *NLK* in osteoblast precursors caused an increase in trabecular bone volume in mice and decreased osteoclast and osteoblast numbers in male and female mice, respectively. The third model, which consists of a conditional deletion of *NLK* in mature osteoblasts, did not show any skeletal phenotype, suggesting that NLK is not required for the function of mature osteoblasts [39].

## 3. NLK and the Immune System

The Wnt signalling pathway is vital for the development and regeneration of most tissues in the human body and is also essential for immune homeostasis with regard to cell differentiation, proliferation and death. Most of the studies investigating immune and blood cells have found canonical Wnt signalling to be integral, since it has been shown to affect parts of haematopoietic stem cell self-renewal, B-cell development in the bone marrow, dendritic cell maturation and T-cell activation and migration [40,41]. Dysregulation of the Wnt signalling pathway in any of these cell types can lead to immunodeficiency, chronic inflammation, and cancer. Furthermore, with regard to T-cell development, the Wnt signalling pathway has been described as an important regulator in all developmental stages of T-cells [42,43,44]. Not until recently was the role of NLK within immune cells investigated, and few studies have used *NLK* transgenic animals to gain better understanding of NLK within the immune system. Recently, we engineered a transgenic mouse model in which Lck-Cre mice were crossed with floxed *NLK* mice to induce a conditional deletion of NLK in the T-cell lineage, including early T-cell progenitor cells. This deletion did not affect the health, white and red blood cells nor lymphoid tissue development of the mice. Nonetheless, *NLK*-deficient mice had a significant reduction in single positive (SP) CD8^+^ thymocytes, without any effects on the SP CD4^+^ thymocyte population. This reduction in SP CD8^+^ thymocytes was caused by an increase in cell death and reduced phosphorylation of LEF1 and HDAC1, suggesting that NLK plays an important role in the survival of CD8^+^ thymocytes [45]. Deletion of *NLK* in regulatory T-cells (Tregs) by using FoxP3Cre mice showed that TAK1 and NLK regulated Treg cell suppressive capacity by modifying the Foxp3 protein. In detail, Foxp3 transcriptional activity was regulated by TAK1-NLK-mediated phosphorylation, and NLK activity was required to maintain homeostatic Treg cell numbers. Furthermore, a colitis mouse model was used to demonstrate that NLK activity is necessary to regulate Treg cell-mediated immunosuppression [46]. *NLK* has also been genetically inactivated in the mouse germ line and the phenotype of this mouse significantly varied with the genetic background of the animal. *NLK*-deficient animals on a C57BL/6 background died in the third trimester of pregnancy, while mice on a 129/Sv background survived up to 4–6 weeks after birth, but displayed a pronounced cerebellar ataxia. On a mixed background, *NLK*-deficient mice showed a severely compromised haematopoietic system, including a reduction in haematopoietic cells, fat tissue in the bone marrow cavity, as well as a loss of bone-lining cells. Furthermore, the lymphoid cell population inside the bone marrow was significantly reduced in *NLK*-deficient animals compared to wild-type animals [47]. The same C57BL/6 × 129/Sv mixed background *NLK*-deficient mouse model described above was used to study the role NLK plays in thymus development. Histological examination of E15.5 *NLK*-deficient thymi revealed a structurally normal thymus that expressed normal Foxn1 levels, thereby suggesting that NLK is not required in the early stages of thymus development [48]. To elucidate the role of NLK in the innate immune response, a conditional myeloid-deficient *NLK* (NLKfl/fl/Lyz2-Cre) mouse model was generated. NLK was shown to play a role in the negative regulation of type I interferon signalling. This mechanism was operated via NLK-mediated phosphorylation of mitochondrial antiviral-signalling protein (MAVS), resulting in its degradation [49].

## 4. NLK and Neuronal Disorders

NLK has been shown to be expressed at high levels in the nervous system and brain; however, its function regarding neurological development and neurodegenerative diseases is not well understood. To study the function of NLK in neurodegenerative diseases, a spinocerebellar ataxias (SCA1) model, which is characterised by progressive ataxia, mild cognitive impairments, speaking and swallowing difficulties, followed by respiratory failure, was selected for analysis. In this model, two independent gene trap insertion lines of murine embryonic stem (ES) cells targeting the *NLK* locus were used to create heterozygous *NLK*-deficient animals that were further crossed with SCA1 knock-in mice. The targeting cassette of the first ES cell line (RRJ297) was inserted into the second intron of the *NLK* gene and the second ES cell line (XN619) was incorporated into intron 1. It was shown that decreased NLK expression might be beneficial against polyglutamine-expanded ATAXIN1 (ATXN1) protein toxicity. Using the *NLK* heterozygous SCA1 knock-in model, it was found that *NLK* haploinsufficiency can rescue SCA1 cerebellar pathology and motor phenotypes [50]. Spinal and bulbar muscular atrophy (SBMA) is an inherited disease resulting in weakness in muscles and degeneration in motor neurons. This neurodegeneration is caused by a specific mutation in the gene encoding the androgen receptor (AR) protein. Mice heterozygous for either of the two gene trap alleles of *NLK* described above were crossed to mice expressing the BAC transgene, which mimics SBMA disease phenotypes, such as muscle atrophy, motor neuron pathology and early lethality. These phenotypes were only observed in male mice due to the dependence of the disease on the expression of AR. Deletion of *NLK* could rescue the muscle atrophy, as well as motor neuron phenotypes, and furthermore extend the lifespan of the heterozygous *NLK* mice compared to control mice. Taken together, NLK expression in mouse delays disease progression but cannot completely prevent SBMA phenotype onset [51]. Huntington’s disease (HD) is caused by a mutation in the huntingtin (HTT) gene encoding mutant huntingtin protein (mHTT) with an expanded polyglutamine tract (PolyQ). The protein clearance system is impaired in HD seen by mHTT aggregation and inclusion in neurons, especially in the striatum and cerebral cortex. *NLK* heterozygous mice generated by the gene trap technology [51] were crossed with HD mouse models to examine the role of NLK in HD. Genetic reduction of NLK worsens HD brain pathology in these mice [52]. In addition, inducing NLK in the knock-out HD models resulted in attenuated brain atrophy and a reduction in mHTT aggregates. Overall, the authors were able to show that NLK has a neuroprotective role in HD, since the deletion of NLK caused accelerated brain dysfunction [52].

## 5. Conclusions

NLK is an evolutionary conserved protein first identified in Drosophila, where it contributes to proper cell movement in the eyes. Importantly, this evolutionary conservation of the NLK protein can be observed from worms to humans, where homology can be observed between human and murine type II NLK protein. Traditionally, NLK was discovered as a negative regulator of the Wnt signalling pathway, but in later years it has been shown to be able to positively regulate Wnt signalling, depending on the organism and developmental processes. Besides Wnt signalling, NLK can affect members of other signalling pathways, such as Notch, NFκB and STAT. In recent years, research has shown that the dysregulation of NLK levels leads to various types of diseases.

**Figure 1:**
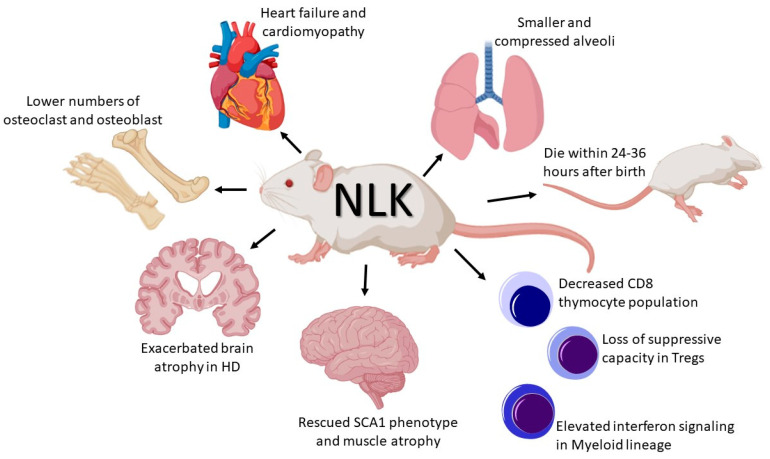
Discoveries in the field of NLK signalling using mouse models. NLK is an evolutionary conserved MAP kinase known to be a negative regulator of the Wnt/β-catenin signalling pathway. Over recent years, mouse models targeting NLK have been established that have led to the discovery of NLK playing an important role in the function of organs including heart, lung, and skeleton. Additionally, multiple breakthroughs have been made showing that NLK regulates the function and differentiation of various immune cells. Finally, evidence has shown that NLK can regulate the phenotype seen in numerous neurodegenerative diseases. Representative summaries are given of major findings regarding the use of mouse models to study the function of NLK signalling. Images in the figure is taken from BioRender.com.

By designing and generating *NLK* knock-out models, it has been discovered that this kinase plays an essential role in the development and function of organs such as the lung, heart and skeleton (see Figure 1). Furthermore, NLK acts as a master regulator for function and differentiation of cells from the immune system, including CD8^+^ SP thymocytes, Treg cells and regulation of the anti-viral response (See Figure 1). Moreover, NLK can regulate neurodegenerative diseases, such as Huntington’s disease and spinocerebellar ataxias (See Figure 1). Mouse studies using *NLK*-deficient mice have generated important knowledge concerning the role of NLK in certain diseases and development. Future ongoing studies will shed light on whether the function of NLK is tissue-dependent and whether NLK can affect the development of other organs and progression of other diseases.

## References

[1] Steinhart Z., Angers S. (2018). Wnt signaling in development and tissue homeostasis. Development.

[2] Sharma R.P., Chopra V.L. (1976). Effect of the Wingless (wg1) mutation on wing and haltere development in Drosophila melanogaster. Dev. Biol..

[3] Nüsslein-Volhard C., Wieschaus E. (1980). Mutations affecting segment number and polarity in Drosophila. Nature.

[4] Nusse R., Varmus H.E. (1982). Many tumors induced by the mouse mammary tumor virus contain a provirus integrated in the same region of the host genome. Cell.

[5] Van Ooyen A., Nusse R. (1984). Structure and nucleotide sequence of the putative mammary oncogene int-1; proviral insertions leave the protein-encoding domain intact. Cell.

[6] Rijsewijk F., Schuermann M., Wagenaar E., Parren P., Weigel D., Nusse R. (1987). The Drosophila homolog of the mouse mammary oncogene int-1 is identical to the segment polarity gene wingless. Cell.

[7] Nusse R., Brown A., Papkoff J., Scambler P., Shackleford G., McMahon A., Moon R., Varmus H. (1991). A new nomenclature for int-1 and related genes: The Wnt gene family. Cell.

[8] Komiya Y., Habas R. (2008). Wnt signal transduction pathways. Organogenesis.

[9] Zhan T., Rindtorff N., Boutros M. (2017). Wnt signaling in cancer. Oncogene.

[10] Latres E., Chiaur D.S., Pagano M. (1999). The human F box protein β-Trcp associates with the Cul1/Skp1 complex and regulates the stability of β-catenin. Oncogene.

[11] Clevers H. (2006). Wnt/β-Catenin Signaling in Development and Disease. Cell.

[12] MacDonald B.T., Tamai K., He X. (2009). Wnt/beta-catenin signaling: Components, mechanisms, and diseases. Dev. Cell.

[13] Coulombe P., Meloche S. (2007). Atypical mitogen-activated protein kinases: Structure, regulation and functions. Biochim. Biophys. Acta (BBA)-Mol. Cell Res..

[14] Choi K.-W., Benzer S. (1994). Rotation of photoreceptor clusters in the developing drosophila eye requires the nemo gene. Cell.

[15] Brott B.K., Pinsky B.A., Erikson R.L. (1998). Nlk is a murine protein kinase related to Erk/MAP kinases and localized in the nucleus. Proc. Natl. Acad. Sci. USA.

[16] Ishitani T., Ninomiya-Tsuji J., Nagai S.I., Nishita M., Meneghini M., Barker N., Waterman M., Bowerman B., Clevers H., Shibuya H. (1999). The TAK1-NLK-MAPK-related pathway antagonizes signalling between beta-catenin and transcription factor TCF. Nature.

[17] Ishitani T., Ninomiya-Tsuji J., Matsumoto K. (2003). Regulation of Lymphoid Enhancer Factor 1/T-Cell Factor by Mitogen-Activated Protein Kinase-Related Nemo-Like Kinase-Dependent Phosphorylation in Wnt/β-Catenin Signaling. Mol. Cell. Biol..

[18] Li M., Wang H., Huang T., Wang J., Ding Y., Li Z., Zhang J., Li L. (2010). TAB2 scaffolds TAK1 and NLK in repressing canonical Wnt signaling. J. Biol. Chem..

[19] Yamada M., Ohnishi J., Ohkawara B., Iemura S., Satoh K., Hyodo-Miura J., Kawachi K., Natsume T., Shibuya H. (2006). NARF, an nemo-like kinase (NLK)-associated ring finger protein regulates the ubiquitylation and degradation of T cell factor/lymphoid enhancer factor (TCF/LEF). J. Biol. Chem..

[20] Ishitani T., Ishitani S. (2013). Nemo-like kinase, a multifaceted cell signaling regulator. Cell. Signal..

[21] Ota S., Ishitani S., Shimizu N., Matsumoto K., Itoh M., Ishitani T. (2012). NLK positively regulates Wnt/β-catenin signalling by phosphorylating LEF1 in neural progenitor cells. EMBO J..

[22] Ohkawara B., Shirakabe K., Hyodo-Miura J., Matsuo R., Ueno N., Matsumoto K., Shibuya H. (2004). Role of the TAK1-NLK-STAT3 pathway in TGF-beta-mediated mesoderm induction. Genes Dev..

[23] Kojima H., Sasaki T., Ishitani T., Iemura S.I., Zhao H., Kaneko S., Kunimoto H., Natsume T., Matsumoto K., Nakajima K. (2005). STAT3 regulates Nemo-like kinase by mediating its interaction with IL-6-stimulated TGFβ-activated kinase 1 for STAT3 Ser-727 phosphorylation. Proc. Natl. Acad. Sci. USA.

[24] Chen J., Lin Q., Ni T., Zhao J., Lin F., Lu X., Lv Y., Ren S., Liu Z., Zhang T. (2020). NLK interacts with 14-3-3ζ to restore the expression of E-cadherin. Oncol. Rep..

[25] Li S.Z., Zhang H.H., Liang J.B., Song Y., Jin B.X., Xing N.N., Fan G.C., Du R.L., Zhang X.D. (2014). Nemo-like kinase (NLK) negatively regulates NF-kappa B activity through disrupting the interaction of TAK1 with IKKβ. Biochim. Biophys. Acta.

[26] Ishitani T., Hirao T., Suzuki M., Isoda M., Ishitani S., Harigaya K., Kitagawa M., Matsumoto K., Itoh M. (2010). Nemo-like kinase suppresses Notch signalling by interfering with formation of the Notch active transcriptional complex. Nat. Cell Biol..

[27] Huang Y., Yang Y., He Y., Li J. (2015). The emerging role of Nemo-like kinase (NLK) in the regulation of cancers. Tumor Biol..

[28] Yasuda J., Tsuchiya A., Yamada T., Sakamoto M., Sekiya T., Hirohashi S. (2003). Nemo-like kinase induces apoptosis in DLD-1 human colon cancer cells. Biochem. Biophys. Res. Commun..

[29] Shaw-Hallgren G., Masoumi K.C., Zarrizi R., Hellman U., Karlsson P., Helou K., Massoumi R. (2014). Association of Nuclear-Localized Nemo-Like Kinase with Heat-Shock Protein 27 Inhibits Apoptosis in Human Breast Cancer Cells. PLoS ONE.

[30] Yang Y., Zhe H., Massoumi R., Ke H. (2019). Decreased expression of nemo-like kinase in melanoma is correlated with increased vascularity and metastasis. Melanoma Res..

[31] Shen N., Duan X.H., Wang X.L., Yang Q.Y., Feng Y., Zhang J.X. (2019). Effect of NLK on the proliferation and invasion of laryngeal carcinoma cells by regulating CDCP1. Eur. Rev. Med. Pharmacol. Sci..

[32] Röttinger E., Croce J., Lhomond G., Besnardeau L., Gache C., Lepage T. (2006). Nemo-like kinase (NLK) acts downstream of Notch/Delta signalling to downregulate TCF during mesoderm induction in the sea urchin embryo. Development.

[33] Thorpe C.J., Moon R.T. (2004). Nemo-like kinase is an essential co-activator of Wnt signaling during early zebrafish development. Development.

[34] Ota R., Kotani T., Yamashita M. (2011). Possible Involvement of Nemo-like Kinase 1 in Xenopus Oocyte Maturation as a Kinase Responsible for Pumilio1, Pumilio2, and CPEB Phosphorylation. Biochemistry.

[35] Cheng X., Liang J., Teng Y., Fu J., Miao S., Zong S., Wang L. (2012). Nemo-like kinase promotes etoposide-induced apoptosis of male germ cell-derived GC-1 cells in vitro. FEBS Lett..

[36] Ke H., Masoumi K.C., Ahlqvist K., Seckl M.J., Rydell-Törmänen K., Massoumi R. (2016). Nemo-like kinase regulates the expression of vascular endothelial growth factor (VEGF) in alveolar epithelial cells. Sci. Rep..

[37] Masoumi K.C., Daams R., Sime W., Siino V., Ke H., Levander F., Massoumi R. (2017). NLK-mediated phosphorylation of HDAC1 negatively regulates Wnt signaling. Mol. Biol. Cell.

[38] Liu R., Khalil H., Lin S.-C.J., Sargent M.A., York A.J., Molkentin J.D. (2016). Nemo-Like Kinase (NLK) Is a Pathological Signaling Effector in the Mouse Heart. PLoS ONE.

[39] Canalis E., Kranz L., Zanotti S. (2014). Nemo-Like Kinase Regulates Postnatal Skeletal Homeostasis. J. Cell. Physiol..

[40] Staal F.J.T., Luis T.C., Tiemessen M.M. (2008). WNT signalling in the immune system: WNT is spreading its wings. Nat. Rev. Immunol..

[41] Chae W.-J., Bothwell A.L.M. (2018). Canonical and Non-Canonical Wnt Signaling in Immune Cells. Trends Immunol..

[42] Ma J., Wang R., Fang X., Sun Z. (2012). β-catenin/TCF-1 pathway in T cell development and differentiation. J. Neuroimmune Pharmacol. Off. J. Soc. Neuroimmune Pharmacol..

[43] Van de Wetering M., de Lau W., Clevers H. (2002). WNT signaling and lymphocyte development. Cell.

[44] Van Loosdregt J., Coffer P.J. (2018). The Role of WNT Signaling in Mature T Cells: T Cell Factor Is Coming Home. J. Immunol..

[45] Daams R., Sime W., Leandersson K., Sitnicka E., Massoumi R. (2020). Deletion of Nemo-like Kinase in T Cells Reduces Single-Positive CD8(+) Thymocyte Population. J. Immunol..

[46] Fleskens V., Minutti C.M., Wu X., Wei P., Pals C.E., McCrae J., Hemmers S., Groenewold V., Vos H.J., Rudensky A. (2019). Nemo-like Kinase Drives Foxp3 Stability and Is Critical for Maintenance of Immune Tolerance by Regulatory T Cells. Cell Rep..

[47] Kortenjann M., Nehls M., Smith A.J., Carsetti R., Schueler J., Koehler G., Boehm T. (2001). Abnormal bone marrow stroma in mice deficient for nemo-like kinase, Nlk. Eur. J. Immunol..

[48] Swann J.B., Happe C., Boehm T. (2017). Elevated levels of Wnt signaling disrupt thymus morphogenesis and function. Sci. Rep..

[49] Li S.Z., Shu Q.P., Song Y., Zhang H.H., Liu Y., Jin B.X., Liuyu T.Z., Li C., Huang X.C., Du R.L. (2019). Phosphorylation of MAVS/VISA by Nemo-like kinase (NLK) for degradation regulates the antiviral innate immune response. Nat. Commun..

[50] Ju H., Kokubu H., Todd T.W., Kahle J.J., Kim S., Richman R., Chirala K., Orr H.T., Zoghbi H.Y., Lim J. (2013). Polyglutamine disease toxicity is regulated by Nemo-like kinase in spinocerebellar ataxia type 1. J. Neurosci..

[51] Todd T.W., Kokubu H., Miranda H.C., Cortes C.J., La Spada A.R., Lim J. (2015). Nemo-like kinase is a novel regulator of spinal and bulbar muscular atrophy. Elife.

[52] Jiang M., Zhang X., Liu H., LeBron J., Alexandris A., Peng Q., Gu H., Yang F., Li Y., Wang R. (2020). Nemo-like kinase reduces mutant huntingtin levels and mitigates Huntington’s disease. Hum. Mol. Genet..

